# Nonlinear Black Phosphorus for Ultrafast Optical Switching

**DOI:** 10.1038/srep43371

**Published:** 2017-02-27

**Authors:** Siam Uddin, Pulak C. Debnath, Kichul Park, Yong-Won Song

**Affiliations:** 1 Center for Opto-Electronic Materials and Devices, Korea Institute of Science and Technology (KIST), Seoul 02792, South Korea; 2 Nanomaterials Science and Engineering, Korea University of Science and Technology, Daejeon 34113, South Korea

## Abstract

The outstanding electronic and optical properties of black phosphorus (BP) in a two-dimensional (2D) but unique single-layer puckered structure have opened intense research interest ranging from fundamental physics to nanoscale applications covering the electronic and optical domains. The direct and controllable electronic bandgap facilitating wide range of tunable optical response coupled with high anisotropic in-plane properties made BP a promising nonlinear optical material for broadband optical applications. Here, we investigate ultrafast optical switching relying on the optical nonlinearity of BP. Wavelength conversion for modulated signals whose frequency reaches up to 20 GHz is realized by four-wave-mixing (FWM) with BP-deposited D-shaped fiber. In the successful demonstration of the FWM based wavelength conversion, performance parameter has been increased up to ~33% after employing BP in the device. It verifies that BP is able to perform efficient optical switching in the evanescent field interaction regime at very high speed. Our results might suggest that BP-based ultra-fast photonics devices could be potentially developed for broadband applications.

In the last few years, 2D crystals emerged as the new flourishing research subjects for their great potential in electronic and optical applications^1^,^2^,^3^,^4^,^5^. The bulk crystals of the 2D semiconducting materials are composed of individual layers in which each layer is vertically stacked by van der Waals force, which characterizes the crystal structure and thus the physical, electronic, and ultimately the optical properties^6^. The evolution of 2D layered material research had drawn the brand new roadmap of nanotechnology, which began with graphene, then was followed by wide-bandgap transition metal dichalcogenides (TMDCs), and was most recently continued with BP^7^,^8^,^9^,^10^. The recent advancements in optical techniques for all-optical switching, signal regeneration, and optical signal control using the 2D materials expanded into photonic and optoelectronic applications, such as serial digital information at ultra-high speed, sensing for atmosphere or biology, quantum computing, electro-optic modulators, and etc^11^,^12^,^13^,^14^. The nonlinear optical effects coupled with the ultrafast optical response of the 2D materials have influenced the design and performance of many advanced high-capacity systems. Indeed, graphene and TMDCs are being used in various applications, ranging from basic electronic research to opto-electronics, photonics, and telecommunications due to their exceptional nonlinear optical properties, especially very high third order susceptibility^2^,^15^. Yet the weak optical absorption with low transistor on-off ratio of graphene due to lack of the bandgap reduce its applicability in semiconducting and optoelectronic devices, on the other hand, limited performance of TMDCs from the high bandgaps render them unsuitable for NIR and m-IR applications. Consequently, a new substitute 2D material is needed that can fill the gap between zero-gap graphene^16^,^17^ and large gap TMDCs^18^,^19^,^20^,^21^ to be integrated into an even wider range of applications.

BP is the most thermodynamically stable allotrope of phosphorus with a monoelemental and anisotropic atomic structure^22^, a layered material whose renaissance as a research focus came after its 2D form was found to have many promises owing to its high carrier mobility, tunable direct bandgap, large on/off ratios of BP-based electronic devices, and anisotropic properties in not only the field of electronics but also optoelectronics and photonics^23^,^24^,^25^,^26^,^27^,^28^,^29^,^30^. Its direct bandgap and electron transition regardless of the number of layers^31^, combined with the wide range layer-dependent tunable bandgap energies that falls between graphene and TMDCs, imply many properties best fit for assorted range of photonic and optoelectronic applications. An attractive feature of BP is the tunable wide bandgap^32^ ranging from ~2 eV for monolayer to ~0.3 eV for bulk by adding successive layers, as a consequence, it can cover a very broad range of electromagnetic spectrum and interact strongly with light. Structurally, inside each individual buckled layer of BP includes two mutually perpendicular in-plane layers which bolsters highly anisotropy in its electronics and optical properties^33^,^34^, hence, merging all the novel electronic and optical properties, BP has become an ideal material for electronics, optoelectronics and photonics.

Recent studies reported BP as a nonlinear optical medium due to the high nonlinear refractive index^35^,^36^ which can be utilized for various nonlinear functional devices for telecommunications, such as wavelength converters, optical switches and signal regenerators. Thus far, the main focus regarding optical nonlinearity has been on saturable absorption^37^,^38^,^39^ of BP which depends on the imaginary part of the 3rd-order nonlinearity, Im(χ^(3)^). On the other hand, the real part, Re(χ^(3)^) is responsible for the nonlinear refractive index change or Kerr effect. FWM, a derivative phenomenon of nonlinear optical Kerr effect, occurs when high power optical signals of two or more different wavelengths are transmitted through a nonlinear optical media. In advanced optics and photonic research, FWM is employed for wavelength conversion, optical parametric amplification, supercontinuum generation, frequency comb generation, and many other signal manipulations^40^,^41^,^42^. Different nonlinear 2D materials such as carbon nanotube^43^, and graphene^44^,^45^ have been demonstrated to be suitable for FWM. However, considering all the novel attributes in electronic and optical domain, nonlinear optical switching e. g., FWM with BP could open a new pathway for conceptually designing applications and systems.

In this paper, a nonlinear optical device employing evanescent field interaction of BP with propagating light has been applied for the FWM demonstration. In the following, we experimentally demonstrate FWM-based wavelength conversion for modulated signals whose frequency ranges up to 20 GHz with a BP-based nonlinear optical device, composed of a side polished fiber, or a D-shaped fiber, deposited with BP. Performance criterion of the device provided an improvement of ~1.6 dB in signal generation with the employment of BP, and thereafter, we carried out an analytical comparison between the optical nonlinearity of a bare optical fiber and that of the BP-deposited D-shaped fiber. Moreover, the wavelength detuning property and nonlinear conversion efficiency of the device were also investigated. Finally, we reiterated the experiment numerous times to verify its high reproducibility, which then indicated the potential of BP in future research for optoelectronics and photonics device fabrications.

## Results and Discussion

### FWM process in BP-deposited fiber

**Figure 1:**
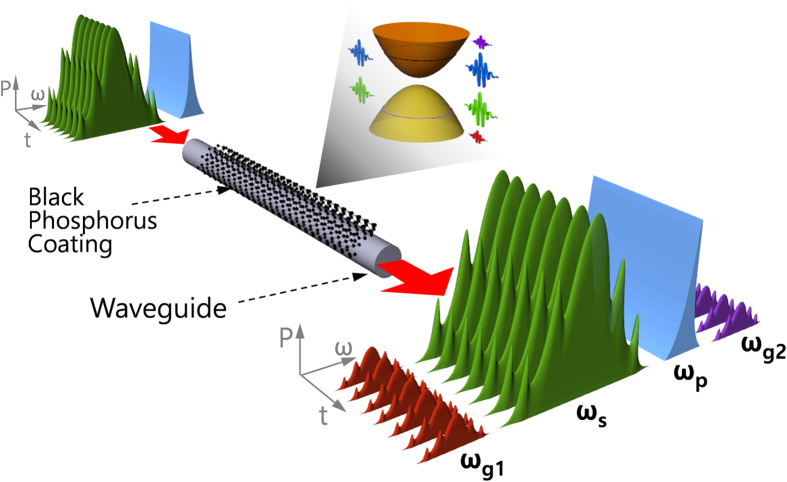
Schematic illustration of FWM-based wavelength conversion in BP–deposited nonlinear fiber optic device. BP is deposited onto the surface of waveguide and consequently generates FWM signals. ω_s_, ω_p_, ω_g1_ and ω_g2_ stand for the frequencies of signal, pump and generated signals, respectively. The signal is modulated with high frequency displaying broadened spectrum, whereas pump is a continuous wave (CW). The generated signals have the exact copies of the modulation information the signal has in ultrafast operating regime. The inset represents the principle of FWM induced and enhanced in the bandgap of BP, where the blue and green wavy signals (left side) represent incoming pump and modulated signal. After scattering and nonlinear frequency generation process the new two generated signals (right side) associate the propagating signals.

The origin of FWM effect lies in the nonlinear response of the optical material through which the optical signals are transmitted. If two optical signals of carrier frequencies ω_s_ and ω_p_ propagate inside a nonlinear medium simultaneously, two more signals of frequency ω_g1_ and ω_g2_ are generated from the third order nonlinear effect, χ^(3)^ of the nonlinear medium. In quantum approach, it is a photonic scattering process during which photons from two or more waves scatter through the medium with third order nonlinearity and give birth to new photons at different frequencies^46^,^47^. One interesting feature of BP is its high optical nonlinearity from which FWM originates. With the BP layers deposited on a D-shaped fiber, a certain portion of the signal propagates outside the fiber, via evanescent field with the extended interaction distance and thus enhances the interaction for the augmented nonlinear effect. Figure 1 is a simplified illustration of mixing two waves with carrier frequencies ω_s_ and ω_p_ co-propagating through BP-deposited nonlinear device, resulting in newly converted signals generated of frequencies ω_g1_ and ω_g2_; where the inset stands for the principle of FWM based nonlinear enhancement in the bandgap structure of BP. From the theory of FWM generation, the electric field^48^ can be expressed as:


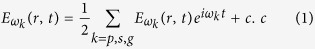


The nonlinear response by the BP deposited evanescent field to the propagating signals lies in the nonlinear polarization induced bound electrons of materials. Considering nonlinear polarization, responsible for generating new signals by FWM process^48^, the total nonlinear response can be defined as:


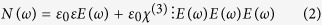


where ε is the relative permittivity, E(ω) is the electric field and the second term in the right side of equation (2) stands for nonlinear polarization. Here, magnitude of the polarization term is governed by the high nonlinear susceptibility from BP.

Generation of FWM based signals thus directly related to the nonlinear response from the device. Due to energy conservation, the frequency of newly generated signals^49^ can be written as:


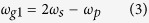



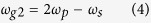


The optical materials for photonic devices require to have an energy band-gap around 0.8 eV^50^ for operating at optical telecommunication band. Since, BP follows a power law^31^


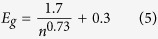


where, n is the number of layer. Hence, considering to the 0.8 eV bandgap, the required layers of BP should be 5–6 that corresponds to ~4.2 nm height of deposited BP layers.

### Device fabrication and characterization

**Figure 2:**
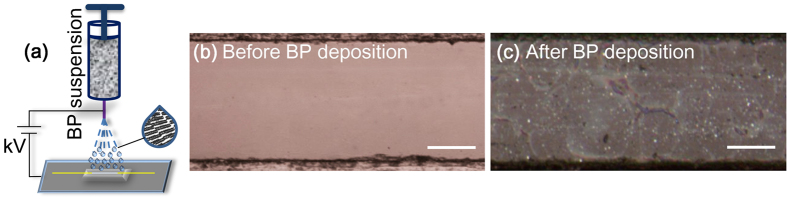
Fabrication of BP deposited on a nonlinear device. (**a**) BP deposition scheme by electrospraying that guarantees a homogeneous spray of BP suspension by splitting the suspension drops into numerous sub-droplets with electrostatic force. OM images of (**b**) side polished bare D-shaped fiber and (**c**) BP-deposited D-shaped fiber; the scale bar is 50 μm.

In order to fabricate the experimental device, a prepared BP suspension was sprayed on the flat surface of a D-shaped fiber via electrospraying, an efficient target-localized homogeneous depositing method already used for different nanomaterials^51^, as schematized in Fig. 2a. The optical microscopic image of the D-shaped fiber before BP deposition is shown in Fig. 2b. Figure 2c portrays the optical image of the sample after the BP deposition, where the BP flakes deposited on the bare fiber can be visibly observed. For an easier characterization of the deposited BP layer, we also simultaneously deposited the BP suspension on a 2 × 2 cm^2^ piece of silica glass using electro-spraying at the same conditions applied to the experimental device.

**Figure 3:**
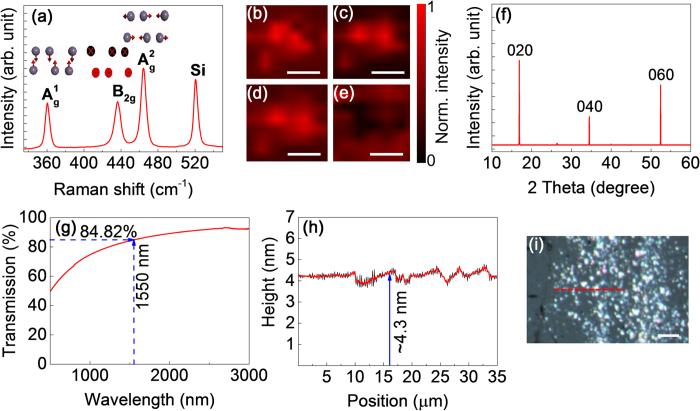
Characterization of the BP layer deposited onto fiber optic device. (**a**) Raman peaks of the deposited BP with different vibration modes. Raman maps of the normalized intensity of (**b**) A^1^_g_, (**c**) B_2g_ and (**d**) A^2^_g_ modes of BP with a scale bar of 2 μm. (**e**) Raman map for the silica peak illustrating the inversion of BP peaks. (**f**) The XRD data of the BP sample. Three major peak correspond to the crystal planes of (020), (040) and (060), respectively. (**g**) The transmission characteristics of the deposited BP flakes with the spectral range from 500 nm to 3000 nm. (**h**) The height profile of the deposited BP. (**i**) OM image of BP-deposited sample. The red dot line represents the part the cross section was analyzed at as shown in (**h**); the scale bar is 10 μm.

Optical Raman spectroscopy was then used to analyze the BP deposited film on the glass. The Raman spectrum of the BP crystal is depicted in Fig. 3a where three peaks corresponding to one out of-plane vibration mode A^1^_g_ and two in-plane vibration modes B_2g_ and A^2^_g_ were observed at 360.4 cm^−1^, 436.1 cm^−1^, 464.4 cm^−1^, respectively. The result agrees with the earlier findings and published ones^52^. Polarization sensitive Raman vibration modes of BP nano-flakes are oriented in-plane^53^ along the substrate and anologous to that of mechanically exfoliated ones^54^. In the next, linearly polarized laser excitation light was used to irradiate the BP deposited sample at normal orientation which leads to the lowest intensity of A^1^_g_ peak^54^. Hence, intensity ratio between the A^1^_g_ and Si peaks can be used to determine the thickness of deposited BP which corresponds to ~5–6 layers^55^ for this specific setup. Again, we calculated the values of A^1^_g_/A^2^_g_ > 0.6 of the Raman spectra which reveal the characteristic of un-oxidized BP on the sample^56^,^57^. The Raman mapping of sample was also carried out for a sample with the area spanning 5 × 5 μm^2^. Raman mapping of characteristic modes A^1^_g_, B_2g_ and A^2^_g_ of BP in Fig. 3b–d indicates the good coverage of the surface by the BP nano-flakes. In contrast, Fig. 3e reveals the Raman map for Silica peak, an inversion of BP peaks in terms of intensity. In addition, XRD was employed to characterize the crystallinity of BP as shown in Fig. 3f. Several distinct diffraction peaks at 16.9°, 34.5°, and 52.4° were observed, corresponding to the (020), (040), and (060) crystal planes respectively, which matched well with the previous report^58^. The weak intensity of (040) diffraction peak might have been originated from distortion in BP crystal by any possible local stress due to randomized orientation of BP flakes, which is however not a degrading factor to optical nonlinearity. We performed a transmission test on the sample for the range from 500 nm to 3000 nm to quantify the absorption level. However, the sample maintained an above 84% transmittance near the communication band used in this experiment. As shown in Fig. 3g, the absorbed light amount (~16%) thus infers that this value corresponds to the thickness of about 5 to 6 layers (the optical absorption of monolayer BP was estimated to 2.8%)^55^. Finally, we obtained a thickness profile of the BP sample by alpha-step measurements as shown in Fig. 3(h,i) and found the thickness of the BP deposition to be up to ~4.3 nm, which is in a strong agreement with the result of the Raman peak analysis. It is worth noting that BP is highly sensitive to oxygen and humidity and thus a thin layer of Polydimethylsiloxane (PDMS) was added on the top of the BP-deposited region to passivate the ambient degradation.

### Experimental observation

**Figure 4:**
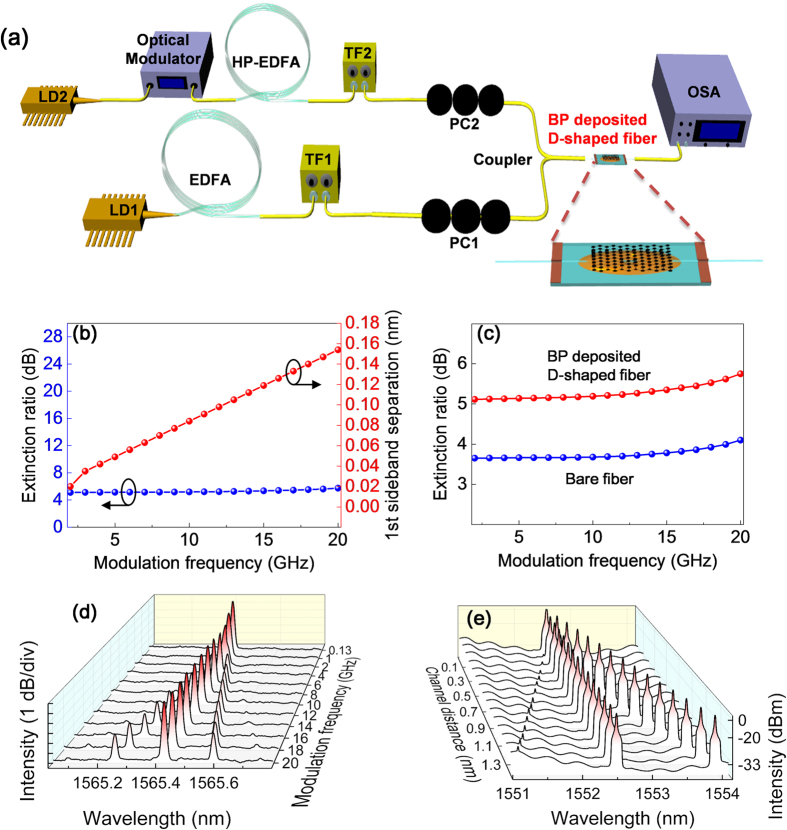
Experimental setup and measured results. ECL: external cavity laser; HP-EDFA: high power erbium-doped fiber amplifier; TF: tunable filter; PC: polarization controller; DC: directional coupler; OSA: optical spectrum analyzer. (**a**) Schematic of experimental setup for the observation of FWM in the BP-deposited device. (**b**) Separation of the first sideband of the newly generated signal. The red trace is for the measured distance from the main peaks to first sidebands, and the blue trace is for the extinction ratio per modulation frequency. (**c**) Efficiency comparison of the generated signals without and with the BP-deposited fiber. (**d**) Modulation frequency (130 MHz to 20 GHz) dependent spectra of the signals generated by the FWM with BP. (**e**) Detuning demonstration of the generated signal by tuning the distance between pump and original signal. Each time the signal and the pump were shifted by 0.1 nm and the generated signal shifted accordingly.

FWM is generated as pump and signal lasers simultaneously propagate through BP deposited an optical nonlinear device, which would be the BP-deposited D-shaped fiber in this experiment. The layout of the experiment is schematized in Fig. 4a where the D-shaped fiber device was used to demonstration FWM effect. In order to demonstrate FWM, the pump wavelength was tuned to 1552.6 nm while the signal wavelength was fixed at 1559 nm. During the experiment, the actual input powers to the BP-deposited D-shaped fiber were estimated to be 24.5 dBm and 20.5 dBm for the pump and signal sources, respectively. However, the input power of the combined laser through the sample was measured at 22.5 dBm.

First, we measured the FWM spectra obtained from a bare D-shaped fiber without BP deposition at different modulation frequencies and found a pair of newly generated signals at 1546.2 nm and 1565.4 nm, originated by the nonlinearity of optical fiber itself. In fact, each newly generated signal carries the same information as the propagating signal with perfect fidelity^48^, signal generated at 1565.4 nm was taken into consideration for further investigation. In order to analyze the nonlinear effect of BP, we replaced the bare D-shaped fiber with the BP deposited one prepared by using electro-spraying, and then measured the generated FWM spectra under the same experimental conditions with different modulation frequencies up to 20 GHz, respectively (Supplementary Information).

When the signal pulses passed through the BP-deposited fiber, its spectral sidebands were gradually separated as the modulation frequency increased, that can be understood with Fourier transformation which allows to visualize this phenomenon in frequency domain while the signals overlap each other in time domain^48^ (Supplementary Information). Figure 4b is a plot of the trace of augmenting distances corresponding to the increased modulation frequency, which is a representation of linear separation change of the sidebands from the main peaks of the newly generated signals. This illustrates that the modulation information of the signal can be successfully copied to the generated signal by the ultrafast operation of BP. Next, we compared the extinction ratio of the signals generated with the bare and BP-deposited D-shaped fiber as illustrated in Fig. 4c. The power of the new signal with BP was observed to be ~1.6 dB higher than that with the bare fiber. Hence, under the same experimental conditions, the generated FWM signal with BP was ~33% stronger in terms of laser power than the one without BP. In the following, the same experiment repeated by slightly shifting the operating wavelength (Supplementary Information) and interchanging the lasers (except the EDFAs), however, the same effect from BP has been found which confirms the operating criteria of our prepared sample within the communication band. Thus, BP had enhanced the FWM generation by contributing to the total nonlinear effect of the device, nevertheless, the total effective nonlinearity of the setup comes from both the silica optical fiber within the setup and the BP on the device.

As BP has several orders higher magnitude^35^ of nonlinearity than that of silica in fiber^59^, the accumulated effective nonlinearity of the setup had increased despite the much shorter interaction distance for BP (~mm scale) than that for silica in fibers (~m scale), thus intensifying the power of the FWM peaks. In Fig. 4d, where all the newly generated FWM signals are depicted. Moreover, during the FWM process, the information carried by the original signal is copied to the converted signals at different wavelengths^60^,^61^,^62^. As a part of the experiment, the trend of signal copying was also demonstrated in the generated signals at the modulation frequencies varying from 130 MHz to 20 GHz. At the low modulation frequency of 130 MHz, the signal remained narrow and so did the converted signal. On the other hand, the converted signal at the modulation frequency of 20 GHz showed the sidebands at relatively same positions as the modulated signal. This phenomenon confirmed that BP can be employed for ultrafast optical signal management. Thereafter, with this experimental setup, we measured the conversion efficiency defined as the power ratio between the generated signal and the original signal, which was estimated as −71.1 dB. Finally, the nonlinearity of BP-deposited fiber device was reaffirmed in terms of the FWM-based wavelength conversion with three different samples. The similar nonlinear response was observed in all three samples, hence the strong reproducibility of the experiment.

Due to the limited system available, both the pump and signal laser were adjusted to fit into one filter with a 4 nm wide bandwidth to demonstrate the detuning process of FWM (Supplementary Information). For detuning, signal was kept fixed at 1552.53 nm and the pump wavelength changed by 0.1 nm each time. As the pump wavelength was shifted by 0.1 nm from the original wavelength, each time the generated signals also shifted by 0.1 nm accordingly, which was another strong evidence of FWM generation. Figure 4e represents the detuning experiment where the newly generated FWM signals shift corresponding to shifting of the pump signal. The detuning study also verified the capacity of the device for bandwidth selection, indicating that the employment of pump signal at either shorter or longer wavelength would result in FWM-based wavelength conversion. The conversion efficiency was measured again, which was −34.3 dB this time as the pump modulated signal and the generated signal were taking place upon the single EDFA.

## Conclusion

In summary, we have demonstrated efficient optical switching by FWM-based wavelength conversion using a BP-deposited fiber optic device. The demonstration has confirmed the optical nonlinearity of BP sufficiently able to perform efficient optical switching for signals with ultrafast modulation frequencies up to 20 GHz. In addition to the electrical advantages of BP such as adjustable bandgap and high on/off ratio, the excellent optical properties of the phosphorus allotrope make it a quite attractive material candidate for the optoelectronic applications that utilizes optical nonlinearity of the media. The confirmation of superior nonlinearity of BP in this study is sure to open new ways to employ the material for future research and commercial uses.

## Methods

### Raman Spectroscopy

The Raman measurements were performed to analyze the deposited BP flakes on silica glass. Raman spectra were recorded on high-resolution Renishaw inVia confocal Raman microscope equipped with a 532 nm laser with 2.0 mW focused on deposited BP flakes using a 50× microscope objective.

### XRD

The XRD patterns were recorded by X-ray diffractometer (Rigaku, D-MAX 2500-PC), equipped with Cu Kα radiation (λ = 1.5406 Å). Data were recorded over a 2θ range of 10–60°, with a step size of 0.04°.

### UV–vis–IR Spectroscopy

The UV–vis–NIR spectroscopy was performed using a VARIAN Cary 5000 UV-Vis-NIR spectrophotometer. This machine covers the wavelength range of 175–3300 nm. We mainly focused on the NIR range, which covers the range of (500 to 3000 nm).

### Experimental Setup

We use our BP-deposited fiber device to demonstrate FWM-based wavelength conversion. In this experiment, the continuous wave (CW) laser from an external cavity laser (ECL1) with a tunable wavelength range of 1525–1575 nm was adopted as the pump laser while another CW laser from the second laser (ECL2) with the fixed wavelength was operated as the signal laser. The CW signal from the ECL2 was first modulated with the carrier frequency (130 MHz to 20 GHz) then amplified by a high power erbium-doped fiber amplifier (HP-EDFA). The amplified signal then passed through a band-pass filter in order to suppress the amplified spontaneous emission (ASE) from the EDFA. The pump signal from the ECL1 was also amplified with a second EDFA then passed through another band-pass filter. Two different polarization controllers (PC) were employed to optimize the state of polarization (SOP) of the both pump and signal. The pump and the modulated signal were then combined by a 3-dB directional coupler (DC), after which the combined laser took part in the FWM generation while propagating through the BP-deposited D-shaped fiber device; the output light spectrum of the sample was measured by an optical spectral analyzer.

## Additional Information

**How to cite this article:** Uddin, S. *et al*. Nonlinear Black Phosphorus for Ultrafast Optical Switching. *Sci. Rep.* **7**, 43371; doi: 10.1038/srep43371 (2017).

**Publisher's note:** Springer Nature remains neutral with regard to jurisdictional claims in published maps and institutional affiliations.

## Supplementary Material

Supplementary Information
