# Supplementary material for: Nonlinear Black Phosphorus for Ultrafast Optical Switching
Source: Sci Rep. 2017 Feb 27;7:43371. doi: 10.1038/srep43371 (PMC5327479; doi:10.1038/srep43371)
Supplement: Supplementary Information [file srep43371-s1.pdf]

Supplementary Information

Nonlinear Black Phosphorus for Ultrafast Optical Switching

Siam Uddin[1,2,*], Pulak C. Debnath[1,2,*], Kichul Park[1], and Yong-Won Song[1,2]

[1]Center for Opto-Electronic Materials and Devices, Korea Institute of Science and Technology (KIST), Seoul 02792, South Korea

[2]Nanomaterials Science and Engineering, Korea University of Science and Technology, Daejeon 34113, South Korea

Correspondence and requests for materials should be addressed to Y.-W.S.

(email: ysong@kist.re.kr)

*These authors contributed equally to this work

Contents

1. **Sample preparation**
2. **Raman peaks dependent thickness measurement**
3. **Comparison of the generated signal with the original signal**
4. **Constant nonlinear enhancement within communication band**
5. **Selectivity of generated signal**
6. **Separation of sidebands**
7. **Detuning experiment**
8. **References**

Sample preparation

Preparing D-shaped fiber: Mid 10 cm of 1 m long single mode fiber (Corning 28) was stripped to make the D-shaped fiber. Stripped optical fibers are fragile and tough to polish without a support. Hence, the stripped fiber was fixed with polyimide tape at the ends by putting it on a slide glass. In the following, epoxy resin was poured on the stripped fiber and heated at 80°C for 30 minutes to get rigid the resin. The D-shaped fiber was then prepared by polishing through the longitudinal axis of that 10 cm segment to a required level. For this experiment, we maintained 1 dB loss per centimeter length. The device was kept under measurement of loss the whole time of polishing to ensure non-cracked and smooth surface of the evanescent field. Finally, the total loss of the device was arisen to ~15 dB after BP deposition by electrospraying.

Preparing BP suspension: The BP powder, prepared by grinding the BP bulk (commercially purchased), was dispersed into dimethylformamide (DMF), an effective solvent for separating and suspending BP for 0.1 mg/mL and ultra-sonicated for 90 min. To remove thick BP particles, the suspension was left standing overnight. The BP suspension was then decanted and centrifuged at 3000 rpm for 10 minutes, and at the end of the centrifugation process, only the BP nanoparticles remained in the DMF solution. Finally, the top 80% of the BP suspension was collected for further experiment.

Electrospraying: The BP suspension was fed into a syringe through a capillary made of stainless steel with a steady speed. For electrospraying, high electric voltage of 10 kV was applied to aerosolize the suspension at the tip of the capillary. The distance between the spraying tip and the D-shaped fiber substrate was set to 15 cm. Deposition times and feeding rates were found to be an adjustable function to obtain desirable thicknesses and homogeneity

of the BP layer. Finally, the BP nanoparticles were recovered upon the D-shaped fiber by drying the device.

Raman peaks dependent thickness measurement

It is possible to measure the thickness of deposited BP on sample from Raman characteristic peaks as the linearly polarized laser was used to irradiate the sample at an orientation of 90° with the BP deposited sample[1]. We measure the intensities of Raman characteristic peaks and silica peaks. We analyze the intensity ratio between the A^1_g peak and the Si peak to calculate thickness in Fig. S1a. Also we analyze the intensity ratio between the A^1_g and the A^2_g peak in Fig. S1b. Absence of spectra with A^1_g /A^2_g <0.6 strongly refers to non-oxidized BP flakes on the substrate[2,3].

Comparison of the generated signals with the original signals

At veracious modulation frequencies, it is shown that the generated signals have the exact copies[4] of the original signals illustrating that the BP layer can manage the high-speed optical signals even in evanescent field interaction regime. Fig. S2,S3 illustrate the insight of copying trend in newly generated signal from propagating signal at low and high modulation frequency, respectively. We also experimented the trend of the generated signal by changing the modulation frequency of the propagating signal gradually. The comparison sets are shown in Fig. S4a,b.

Constant nonlinear enhancement within communication band

The same experiment (discussed detail in the manuscript) was performed by tuning the pump and signal at 1544.9 nm and 1551.9 nm respectively. Newly generated signals were found at position 1537.9 nm and 1558.9 nm with similar extinction ratio enhancement by using BP.

This phenomena confirms that the nonlinearity enhancement was same for similar operating criteria within communication band. This experiment was also performed with low and high modulation frequency can be easily understood with Fig. S5a,b.

Selectivity of generated signal

The newly generated signal at 1546.2 nm was compared for both the BP-deposited and without BP cases, illustrated in Fig. S6a,b. The analogous nonlinear enhancement was found for the case for BP deposited fiber which allows to select either of the generated signals as both of carry the same information as the propagating signal with perfect fidelity[4].

Separation of sidebands

Mathematically, extending sideband distance due to incremental modulation frequency can be understood by following equation in terms of wavelength[5]:

$$D = \frac{\lambda^2 f_m}{c} \tag{1}$$

Where D, λ, f_m and c stand for separation distance, wavelength, modulation frequency, speed of light, respectively. This equation relates the separation distance and modulation frequency in a linear fashion.

In case of FWM, the newly converted signals are generated only in the case when two or more (Pump and modulated signal in this experiment) signals simultaneously propagate and overlap together in time domain[4].

Because of many spectral components in each of the propagating signals, it is hard to distinguish the generated sidebands from the signal spectra in time domain at narrow bandwidth. Hence, Fourier transform can be adopted to understand the gradual separation of sidebands from the main peak of the newly generated signal.

Detuning experiment

In order to tune the wavelength of the generated signals within the same EDFA spectral range, only a single EDFA is used and the signals are detuned by change the wavelength of pumps. The experimental setup scheme and optical spectra are schematized in Fig. S7a-c.

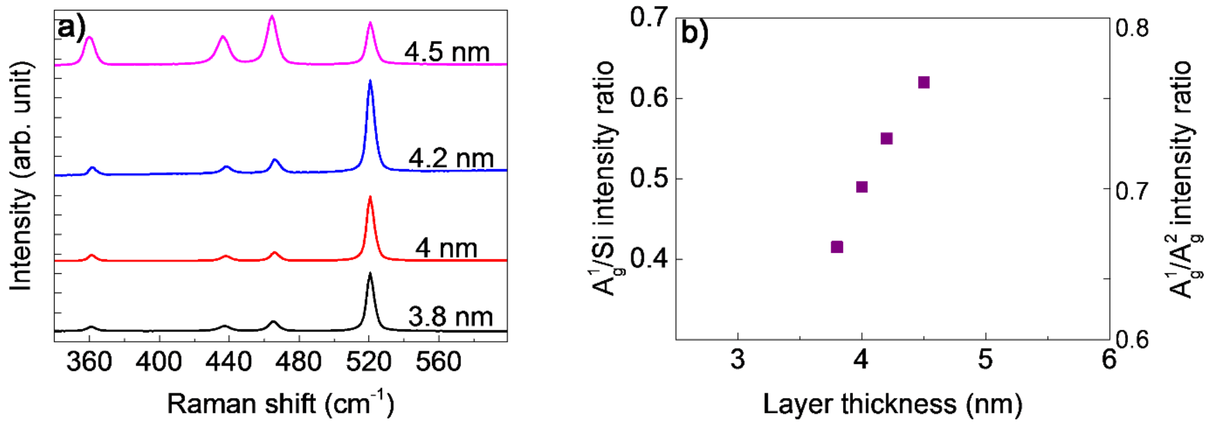

Figure S1. Raman characterization of deposited BP as a function of thickness. (a) Raman analysis of the BP layers with the thickness ranging from 3.5 ~ 4.5 nm. (b) Integrated ratio of A^1_g/Si and A^1_g /A^2_g with respect to the layer thickness.

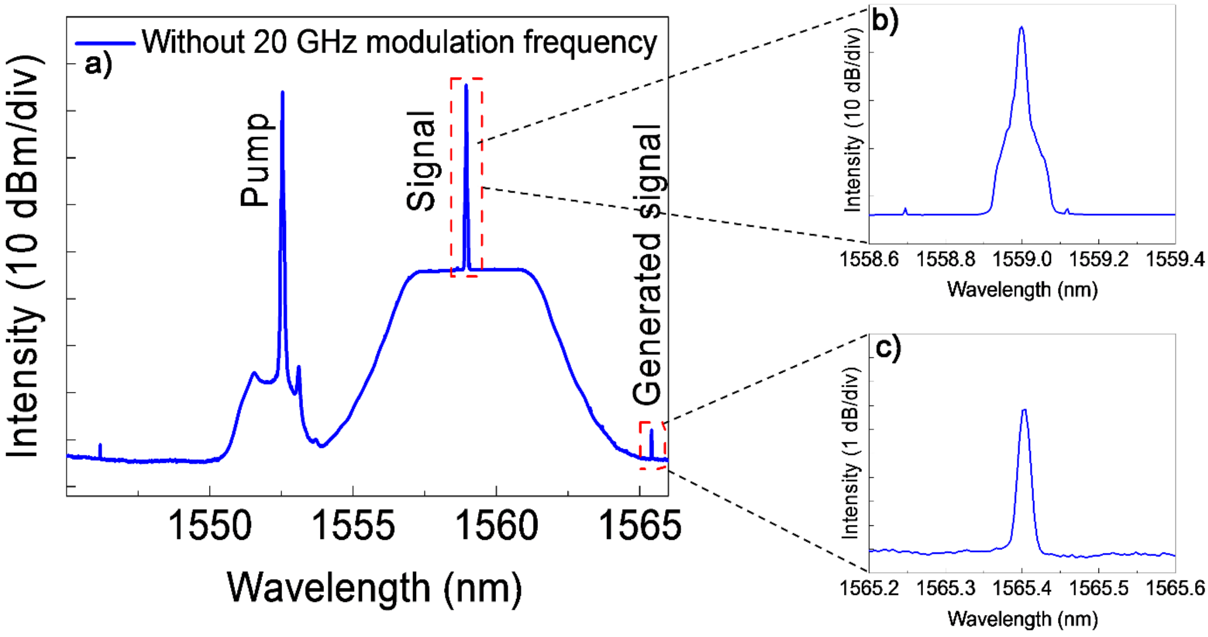

Figure S2. Copying trend of generated signal at lower modulation frequency. (a) Full range spectral illustration of pump, signal and generated signal without high modulation frequency (b) Magnified view of signal only. (c) Magnified view of generated signal.

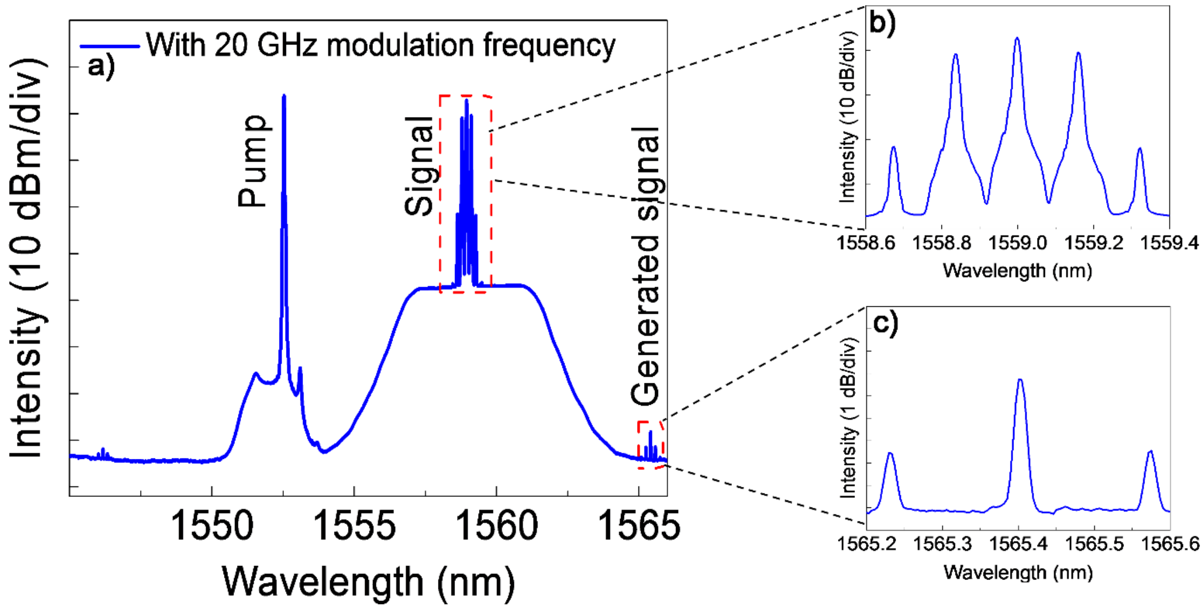

Figure S3. Copying trend of generated signal at higher modulation frequency. (a) Full range spectral illustration of pump, signal and generated signal with high modulation frequency (b) Magnified view of signal only. (c) Magnified view of generated signal.

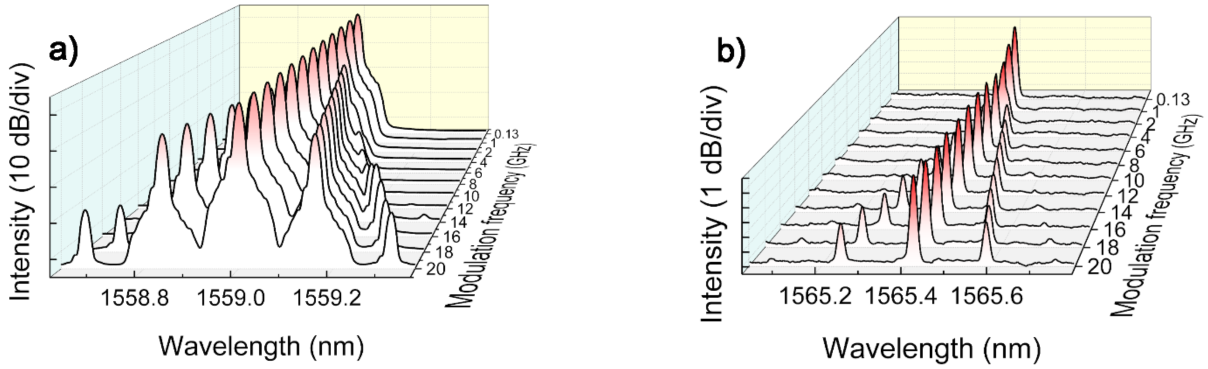

Figure S4. Illustration of modulated and generated signals. (a) Modulated signals at successively higher modulation frequencies. (b) Generated signals corresponding to the modulated signals.

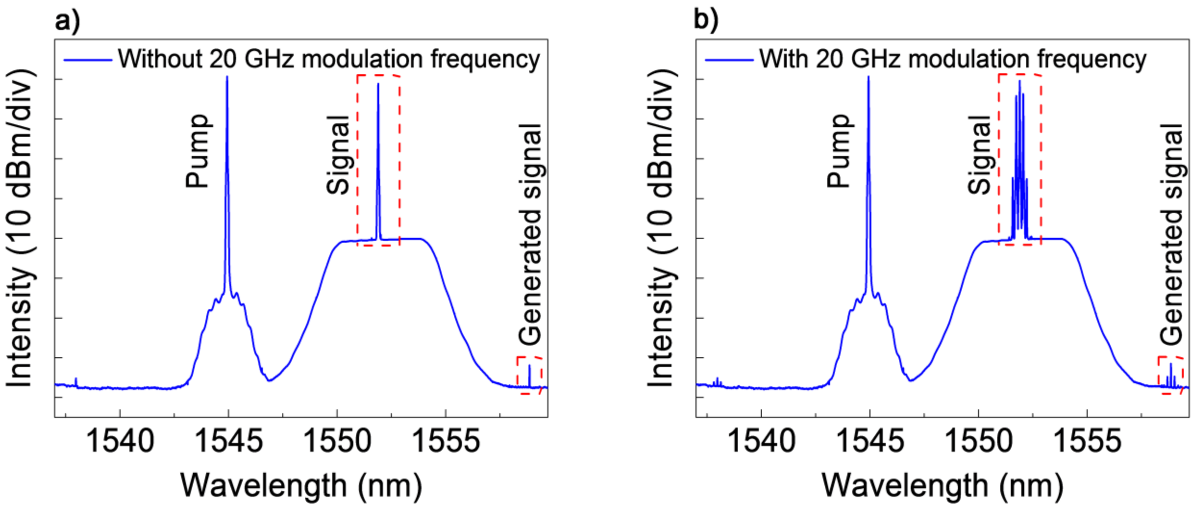

Figure S5. Similar nonlinear enhancement within the communication band with modulation frequency (a) 130 MHz and (b) 20 GHz, respectively.

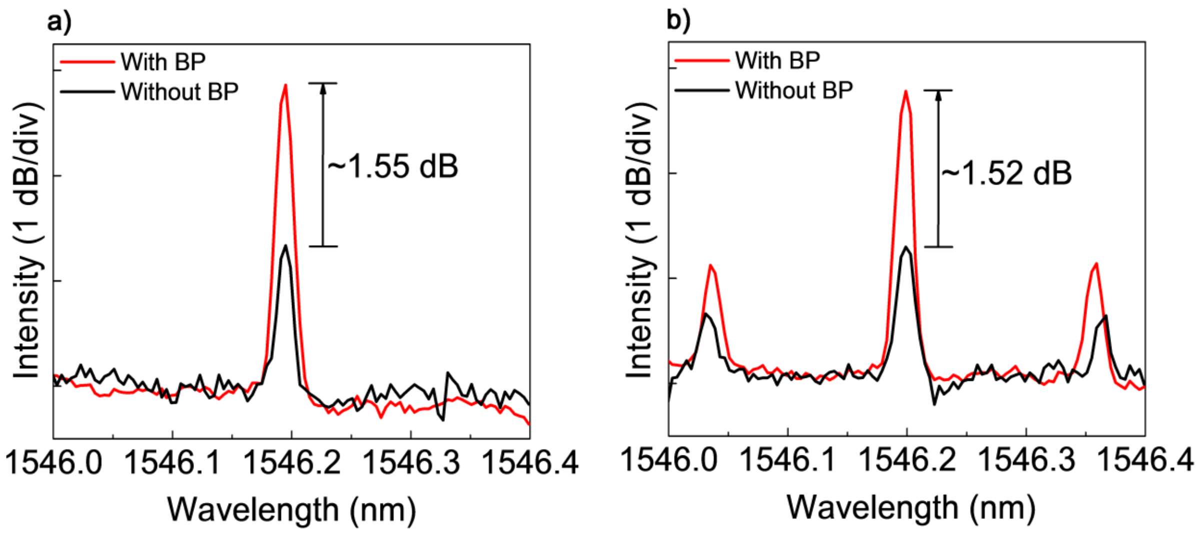

Figure S6. Comparison of extinction ratio between with BP and without BP deposition case at different modulation frequency (a) 130 MHz and (b) 20 GHz, respectively.

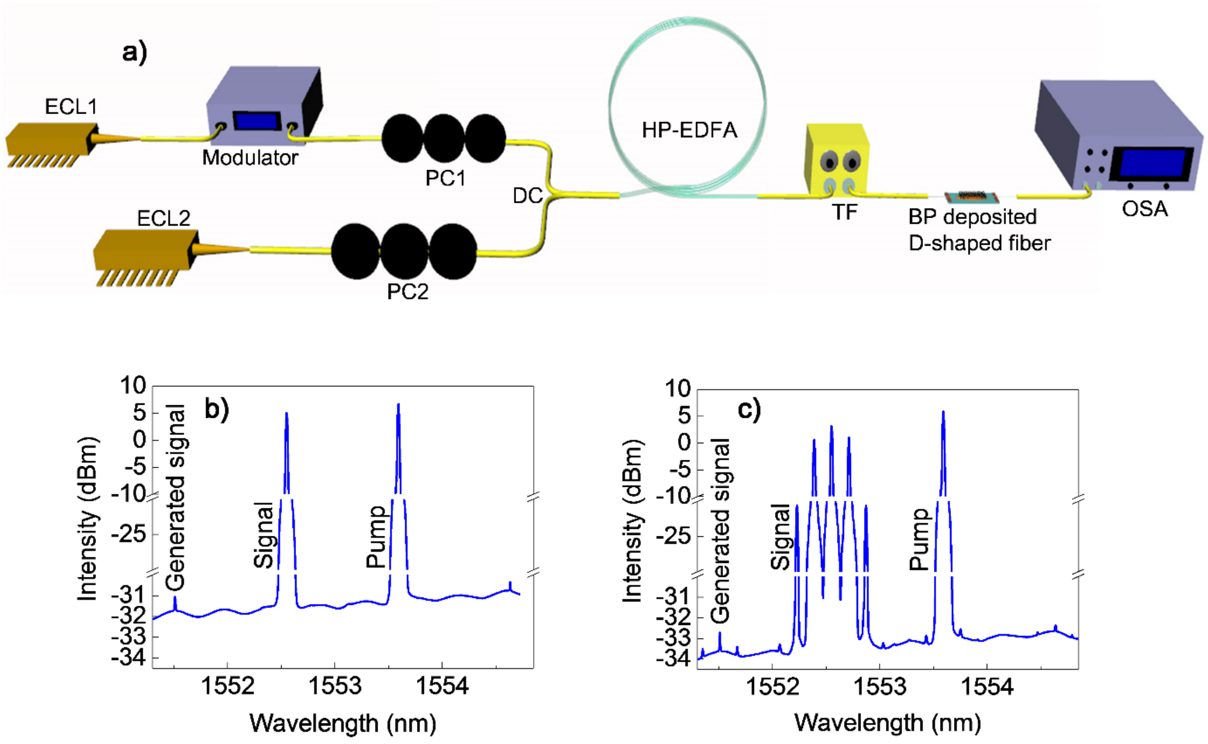

Figure S7. Detuning experiment. (a) System setup for detuning experiment. Full range spectral illustration for all four-wave-mixing (FWM) channels with the signals modulated at (b) 130 MHz and (c) 20 GHz, respectively.

References

S1. Yasaei, P. *et al.* High-quality black phosphorus atomic layers by liquid-phase exfoliation. *Adv. Mater.* **27**, 1887–1892 (2015).

S2. Favron, A. *et al.* Photooxidation and quantum confinement effects in exfoliated black phosphorus. *Nat. Mater.* **14**, 826–832 (2015).

S3. Hanlon, D. *et al.* Liquid exfoliation of solvent-stabilized few-layer black phosphorus for applications beyond electronics. *Nature Commun.* **6**, 8563 (2015).

S4. Agrawal, G. P. *Nonlinear Fiber Optics* (Academic, San Diego, 2007).

S5. Eriksen, G. Oscillation broadening of lines from ionized elements in stellar spectra. *Astrophys. Norv.* **8**, 1 (1961).
